# Draft Whole-Genome Sequences of 10 Atypical Enteropathogenic Escherichia coli Strains Isolated in Brazil

**DOI:** 10.1128/MRA.01432-18

**Published:** 2018-12-06

**Authors:** Fernanda F. Santos, Emily J. Richardson, Jack A. Bryant, Denise Yamamoto, Waldir P. Elias, Tânia A. T. Gomes, Ian R. Henderson

**Affiliations:** a Departamento de Microbiologia, Imunologia e Parasitologia, Escola Paulista de Medicina, Universidade Federal de São Paulo, São Paulo, Brazil; b Institute of Microbiology and Infection, College of Medical and Dental Sciences, University of Birmingham, Birmingham, United Kingdom; c Instituto Butantan, Laboratório de Bacteriologia, São Paulo, Brazil; Georgia Institute of Technology

## Abstract

The number of diarrhea cases caused by atypical enteropathogenic Escherichia coli (aEPEC) has been increasing worldwide. Here, we report the draft whole-genome sequences of 10 aEPEC strains isolated in Brazil. These sequences will provide an important source for future studies concerning aEPEC pathogenicity and genetic markers of potentially virulent strains.

## Abstract (precis)

The number of diarrhea cases caused by atypical enteropathogenic Escherichia coli (aEPEC) has been increasing worldwide. Here, we report the draft whole-genome sequences of 10 aEPEC strains isolated in Brazil.

## ANNOUNCEMENT

Globally, diarrheal diseases are the second leading cause of death and the leading cause of malnutrition in children under 5 years old (1, 2). One of the most significant etiological agents of moderate-to-severe diarrhea is enteropathogenic Escherichia coli (EPEC), which is one of the diarrheagenic E. coli pathotypes. EPEC is subdivided into typical EPEC (tEPEC) and atypical EPEC (aEPEC) based on the presence of the EPEC adherence factor (EAF) plasmid in the former group and its absence in the latter group (3, 4).

The main pathogenic mechanism of both tEPEC and aEPEC is the formation of attaching and effacing (AE) lesions in the intestinal epithelium, in which intimate adherence between bacteria and host cells occurs (5–8). The genes required for the establishment of AE lesions are located on the locus of enterocyte effacement (LEE) island, which is approximately 35 kb long (9).

The number of cases of diarrhea caused by aEPEC has increased even in industrialized countries. This information reveals the need for a thorough knowledge of the genetic traits of aEPEC organisms and their mechanisms of pathogenicity in order to develop appropriate control strategies (4, 10, 11).

The 10 aEPEC strains we sequenced were isolated from feces of children under 5 years old during epidemiological studies carried out in São Paulo, Brazil (1985 to 1986 and 1989 to 1990) by the Laboratory of Microbiology of the Universidade Federal de São Paulo (UNIFESP) and in Salvador (2003 to 2004) by the Laboratory of Bacteriology of the Butantan Institute (12, 13). All the strains were stored at –80°C in lysogeny broth (LB) and 15% glycerol and were routinely cultured in LB for 18 to 20 h at 37°C.

DNA extraction was performed using the QIAamp DNA minikit (Qiagen) according to the manufacturer’s instructions. DNA libraries from each aEPEC genome were prepared using the Nextera XT library prep kit (Illumina, San Diego, CA) and sequenced at MicrobesNG (University of Birmingham, UK) on the Illumina HiSeq 2500 platform using 2- × 250-bp paired-end reads, achieving between 34 and 157× depth (Table 1). Trimmomatic version 0.30 (14) was used to adapt and quality trim the reads with a sliding window quality cutoff of Q15. The *de novo* assembly and annotation of the genomes were performed using the software SPAdes version 3.9.1 (with the –careful flag) (15) and Prokka version 1.12 (16), respectively. The genome assembly metric, calculated using QUAST, and the number of coding sequences (CDS) annotated in each aEPEC genome are shown in Table 1. Unless specified, all software was used with default settings.

**TABLE 1:** Genome assembly metrics and number of CDS of 10 aEPEC genomes

Strain	Mean coverage (×)	No. of contigs	Largest contig (bp)	Total length (bp)	GC content (%)	*N*_50_ (bp)	*N*_75_ (bp)	*L*_50_ (contigs)	*L*_75_ (contigs)	No. of CDS	GenBank accession No.
1331-2	141.587	148	428,643	5,187,915	50.61	112,592	63,355	14	29	4,998	QYVD00000000
2012-1	78.7493	230	247,360	5,607,608	50.43	97,093	41,042	21	43	5,482	QYVC00000000
2531-13	157.673	265	242,288	5,055,689	50.59	73,162	36,314	21	45	4,843	QYVB00000000
3522-6	85.7236	144	317,272	4,730,622	50.57	97,356	51,903	15	32	4,494	QYVA00000000
3881-3	109.173	73	359,010	4,701,486	50.72	241,425	130,938	9	15	4,442	QYUZ00000000
3991-1	34.3279	239	196,577	4,812,795	50.79	73,367	32,807	22	46	4,569	QYUY00000000
4581-2	87.0432	78	406,594	4,696,360	50.41	136,790	95,011	11	20	4,382	QYYF00000000
4632-3	93.7625	174	288,111	4,855,364	50.6	90,810	36,963	16	38	4,617	QYUX00000000
BA2103	85.4008	252	285,340	5,412,725	50.48	102,510	48,630	18	36	5,231	QYUW00000000
BA4095	43.121	71	510,385	4,797,996	50.61	216,769	124,397	8	14	4,568	QYUV00000000

These sequences will provide an important source for futures studies concerning aEPEC pathogenicity and genetic markers of potentially virulent strains. Furthermore, these data will permit comparative studies to be carried out with aEPEC strains isolated in Brazil and others isolated in different countries.

### Data availability.

The reads used for assembly of the 10 aEPEC genomes were deposited in the Sequence Read Archive (SRA) at the National Center for Biotechnology Information (NCBI) under the accession number PRJNA490882, and the whole-genome shotgun sequences were deposited in the GenBank database under the accession numbers shown in Table 1. The versions described in this paper are the first versions.
